# Bacteria, Fungi, and Enzymes in Soil Treated with Sulcotrione and Terbuthylazine

**DOI:** 10.3390/ijms241914469

**Published:** 2023-09-23

**Authors:** Małgorzata Baćmaga, Jadwiga Wyszkowska, Agata Borowik, Jan Kucharski

**Affiliations:** Department of Soil Science and Microbiology, Faculty of Agriculture and Forestry, University of Warmia and Mazury in Olsztyn, 10-719 Olsztyn, Poland; m.bacmaga@uwm.edu.pl (M.B.); agata.borowik@uwm.edu.pl (A.B.); jan.kucharski@uwm.edu.pl (J.K.)

**Keywords:** herbicides in soil, microorganisms, metagenomics, enzymatic activity, *Zea mays* L.

## Abstract

Soil’s biological equilibrium, disturbed by the uncontrolled penetration of pesticides, can be restored by the activity of native microorganisms, which show abilities in neutralizing these xenobiotics. Therefore, this research is necessary in the search for new microorganisms used in the process of the bioremediation of contaminated soils. The aim of this study was to evaluate the effects of the herbicides, Sulcogan 300 SC, Tezosar 500 SC, and Sulcotrek 500 SC, applied to soil at the manufacturers’ recommended dosage as well as 10-fold higher, on the abundance of microorganisms, the diversity and structure of bacterial and fungal communities, the activity of soil enzymes, and the growth and development of *Zea mays* L. It was found that herbicides in contaminating amounts stimulated the proliferation of organotrophic bacteria and inhibited the growth of fungi. Organotrophic bacteria and actinobacteria were represented by K-strategies and fungi by r-strategies. Bacteria belonging to the phylum, *Actinobacteriota*, represented by the genus, *Cellulosimicrobium*, were most abundant in the soil, while among the fungi, it was the phylum, *Ascomycota*, represented by the genus, *Humicola* and *Chaetomium*. The herbicides decreased urease activity while increasing arylsulfatase and acid phosphatase activity. They had a positive effect on the growth and development of *Zea mays* L., as evidenced by an increase in the values of the plant tolerance index (TI) and the maize leaf greenness index (SPAD). The results indicate that soil microorganisms and enzymes are suitable indicators reflecting the quality of herbicide-treated soil.

## 1. Introduction

In order to protect the environment and preserve the biodiversity of organisms, special attention is paid to the neutralization of pollutants by means of biological methods, which are not only cost-effective and efficient, but also safe for the environment. These methods involve, in particular, the use of microorganisms that convert harmful xenobiotics into non-toxic compounds [1]. A safe and healthy natural environment is essential for life on Earth. However, its proper functioning has been disrupted by anthropogenic activity, which has led to the introduction of toxic and dangerous substances, including pesticides, into the environment [2]. Pesticides are heterogeneous chemical compounds that are widely used in agriculture and horticulture, mainly to eradicate weeds, fungi, and insects. Once applied, more than 98% of these xenobiotics are suspected to reach organisms other than their target organisms, thus posing a threat to the natural environment [3]. These chemicals pervade the environment, mainly as a result of their absorption by plants or accumulation in the soil environment. Due to their phytotoxic influence on plants sensitive to their effects, they contribute to a delay in flowering and a reduction in yields of many crops [4]. The effects of pesticides on the soil ecosystem entail the response of soil microorganisms and enzymes, and the magnitude of these effects depends on their physicochemical properties (molecular weight or lipophilicity) as well as on the properties of soil (soil texture and structure, pH, temperature, humidity, adsorption capacity, and biological activity) [5]. In addition, Tonello et al. [6] have shown that the ability of these chemicals to migrate in soil is also influenced by the partition coefficient (K_ow_).

The health of soil primarily entails its ability to promote plant growth and development and animal health, and to maintain or improve water and air quality [7]. The health and fertility of soil depend on its type and bioactivity and on microbial abundance and diversity [8]. Soil microorganisms represent an integral part of soil ecosystems, which play a very important role in the degradation and formation of soil minerals and organic matter, the formation of soil aggregates, the circulation of elements in the biosphere, the provision of nutrients, and the formation of soil [9,10]. The numbers, diversity, and structure of microbial communities reliably reflect changes induced in soil by stress factors. Thus, these parameters can be successfully used to determine the quality and stability of the soil environment [11]. Li et al. [12] have emphasized that the stability of soil ecosystems can be disturbed by uncontrolled and wrong use of pesticides. Inadequate management of soil resources can directly affect the physicochemical properties of the soil and thereby, also the soil microorganisms [13,14], the growth and diversity of which are closely linked to soil fertility [15]. Li et al. [16] have indicated that the abundance, diversity, and structure of microbial communities are significantly correlated with soil pH and moisture, and soil enzyme activity. In addition, changes in soil microbiological properties may be due a reduction in organic carbon, total nitrogen, total phosphorus, and total potassium contents in the soil [17].

In soils, pesticides are degraded primarily through co-metabolism, as well as hydrolysis and photolysis. During the co-metabolism process, microorganisms use the active substances of pesticides as a source of carbon or energy, and also as a second nutrient that provides them with energy. This process is aided by many enzymes secreted by microorganisms [18]. In turn, chemical hydrolysis leads to the formation of intermediate substances, which are then degraded by microorganisms as a result of biochemical reactions [19]. One of the most effective ways to neutralize pesticides is to degrade them via microorganisms that exhibit remarkable catabolic potential due to their genes and the enzymes they secrete, e.g., microorganisms belonging to the *Alcaligenes, Cellulosimicrobium*, *Microbacterium*, *Micrococcus*, *Methanospirillum*, *Aeromonas*, *Sphingobium*, *Flavobacterium*, *Rhodococcus*, *Aspergillus*, *Penecillium*, *Trichoderma*, *Streptomyces*, *Rhodotorula*, *Candida*, and *Aureobasidium* genera [20,21,22]. The most commonly isolated enzymes produced by microorganisms that are involved in pesticide degradation are oxidoreductases, monooxygenases, dioxgenases, phosphotriesterases, haloalkane, and dehalogenases. The genes coding pesticide-degrading enzymes are *Opd* (*Pseudomonas diminuta*), *opaA* (*Alteromonas* spp.), *opdA* (*Alteromonas radiobacter*), *adpB (Nocardia* spp.), *pepA* (*Escherichia coli*), *hocA* (*Pseudomonas monteilli*), *pehA* (*Burkholderia caryophilli*), *Phn* (*Bacillus cereus*), *ophB* (*Burkholderia* spp. JBA3), *ophC2* (*Stenotrophomonas* spp. SMSP-1), *OpdB* (*Lactobacillus brevis*), *Imh* (*Arthrobacter* spp. scl-2), *Mpd* (*Ochrobactrum* spp. Yw28, *Rhizobium radiobacter*), *Oph* (*Arthrobacter* spp.), *Mph* (*Arthrobacter* spp. L1), *MpdB* (*Burkholderia cepacia*), *opdE* (*Enterobacter* spp.), *A-opd* (*Aspergillus niger*), *P-opd* (*Penicillium lilacinum*) [23]. In turn, Bass and Field [24] have emphasized the enormous potential of the bacteria of the genus, *Bacillus*, for pesticide biodegradation, due to the production of many extracellular enzymes (e.g., laccase, esterase, hydrolases, carboxylesterases, and organophosphate hydrolase), which exhibit a broad range of effects on the breakdown of many pesticide compounds [25]. High degradation potential has also been observed in the case of *Pseudomonas* genus bacteria, due to their rapid adaptation in a pesticide-contaminated environment. In addition, these bacteria secrete enzymes involved in the conversion of these chemical compounds, i.e., oxidoreductases, dioxins, cytochrome P450, carboxylases, and phosphotriesterases [26]. Mollea et al. [27] have claimed that fungi function better in a polluted environment than bacteria. To biodegrade pesticides, they use oxidases and peroxidases [28], causing structural changes in the molecule of these chemicals, making them non-toxic [29]. Microorganisms use contaminants as a source of nutrients for their growth and basic metabolic functions; thereby, many microbial populations can survive in contaminated environments. Determination of the effects of pesticides on soil microbiota requires multi-faceted studies and a multi-step approach [2]. There have been many investigations addressing the effects of herbicides on the natural environment, but they are still incomplete and require extending the knowledge of these chemicals. It is very important to understand the interaction between soil organisms and herbicides introduced into the soil. Therefore, we hypothesized that the herbicides tested can be considered environmentally friendly as long as they cause no changes in the diversity and structure of microorganisms, soil biochemical activity, as well as the growth and development of maize. In order to verify the research hypothesis, the aim of the study was to assess changes taking place in the soil environment under the influence of three herbicides (Sulcogan 300 SC, Tezosar 500 SC, and Sulcotrek 500 SC), with particular emphasis put on microbiological parameters (abundance, diversity of bacteria, and fungi), biochemical parameters (activity of dehydrogenases, catalase, alkaline phosphatase, acid phosphatase, *β*-glucosidase, arylsulfatase, and urease), and phytotoxic parameters (dry matter yield, length of aboveground parts, and leaf greenness index of maize).

## 2. Results

### 2.1. Response of Soil Microorganisms to Herbicides

The study has shown that the tested herbicides stimulated the growth of organotrophic bacteria, regardless of the dose used. Tezosar 500 SC applied to the soil in the RD dose caused an increase in the number of fungi, whereas Sulcogan 300 SC and Sulcotrek 500 SC, the herbicides used in the 10RD dose, reduced their number. The number of actinobacteria increased under the influence of Sulcogan 300 SC and Tezosar 500 SC administered into the soil in the RD dose and under the influence of Sulcotrek 500 SC applied in the 10RD dose, but decreased in the soil treated with Tezosar 500 SC in the 10 RD dose and Sulcotrek 500 SC in the RD dose (Table S1).

The significant effect of herbicides on the microbial abundance was confirmed by the values of the IF index (Table 1). The study demonstrated a positive effect of the analyzed herbicides on the number of organotrophic bacteria, regardless of their dose (IF ranged from 0.074 to 0.492). The actinobacteria responded negatively to Tezosar 500 SC added to the soil in the 10 RD dose (IF = −0.128) and to Sulcotrek 500 SC applied in the RD dose (IF = −0.135). The most sensitive to the tested herbicides applied to the soil at a dose of 10RD turned out to be fungi, as evidenced by negative values of the IF index. In the case of the Sul10RD soil sample, the IF index reached −0.175, whereas it was −0.105 for Tez10RD and −0.209 for Suc10RD.

**Table 1:** Index of the impact (IF) of herbicides on number of soil microorganisms.

Object	Org	Act	Fun
SulRD	0.314 ± 0.100 ^ab^	0.164 ± 0.027 ^b^	0.026 ± 0.006 ^bc^
Sul10RD	0.074 ± 0.077 ^c^	0.026 ± 0.028 ^c^	−0.175 ± 0.023 ^d^
TezRD	0.421 ± 0.122 ^a^	0.279 ± 0.077 ^ab^	0.254 ± 0.031 ^a^
Tez10RD	0.145 ± 0.074 ^bc^	−0.128 ± 0.046 ^d^	−0.105 ± 0.030 ^cd^
SucRD	0.342 ± 0.042 ^a^	−0.135 ± 0.085 ^d^	0.087 ± 0.133 ^b^
Suc10RD	0.492 ± 0.012 ^a^	0.367 ± 0.007 ^a^	−0.209 ± 0.023 ^d^

Sul—Sulcogan 300 SC; Tez—Tezosar 500 SC; Suc—Sulcotrek 500 SC; RD—dose recommended by the manufacturer; 10RD—dose 10-fold higher than recommended by the manufacturer; Org—organotrophic bacteria; Act—actinobacteria; Fun—fungi; ±—standard deviation. Homogeneous groups denoted by letters (^a–d^) were calculated separately for each group of microorganisms.

The colony development index (CD) presented in Table 2 provides information on the growth rate of the microorganisms. The data obtained indicate that r-strategist fungi developed in soil treated with herbicides, with the greatest changes in their CD value were recorded in the soil treated with Tezosar 500 SC, as their CD decreased from 41.762 (control soil) to 37.105 (RD dose) and 35.877 (10RD dose). Organotrophic bacteria and actinobacteria were represented by K-strategists. The tested preparations reduced the CD value of organotrophic bacteria compared to the control soil, except for the soil treated with Tezosar 500 SC in the RD dose (wherein the CD value was at the same level as in the control soil). In the case of the actinobacteria, herbicides caused no significant changes in their CD index, except for the soil amended with Sulcogan 300 SC in the 10RD dose, where its value decreased from 23.436 to 20.441.

**Table 2:** Effect of herbicides on microbial colony development index (CD).

Object	Org	Act	Fun
C	31.390 ± 1.005 ^a^	23.436 ± 0.324 ^a^	41.762 ± 0.127 ^a^
SulRD	29.567 ± 0.311 ^b^	24.065 ± 0.564 ^a^	38.886 ± 0.600 ^bc^
Sul10RD	28.528 ± 0.469 ^b^	20.441 ± 0.138 ^b^	37.105 ± 0.871 ^cd^
TezRD	31.448 ± 0.073 ^a^	23.173 ± 0.096 ^a^	35.877 ± 0.470 ^d^
Tez10RD	28.520 ± 0.361 ^b^	23.350 ± 1.043 ^a^	31.758 ± 1.552 ^e^
SucRD	29.090 ± 0.715 ^b^	23.962 ± 0.247 ^a^	39.801 ± 0.984 ^b^
Suc10RD	28.736 ± 0.297 ^b^	23.546 ± 0.127 ^a^	39.133 ± 0.908 ^bc^

C—control soil; Sul—Sulcogan 300 SC; Tez—Tezosar 500 SC; Suc—Sulcotrek 500 SC; RD—dose recommended by the manufacturer; 10RD—dose 10-fold higher than recommended by the manufacturer; Org—organotrophic bacteria; Act—actinobacteria; Fun—fungi; ±—standard deviation. Homogeneous groups denoted by letters (^a–e^) were calculated separately for each group of microorganisms.

Herbicides also significantly affected the values of the ecophysiological diversity (EP) index of microorganisms (Table 3). In the soil treated with Sulcogan 300 SC in RD and 10RD does, with Tezosar 500 SC in the 10RD dose, and with Sulcotrek 500 SC in the RD dose, there was an increase in the EP values determined for organotrophic bacteria. The herbicides did not cause significant changes in the EP index of the actinobacteria, except for the soil treated with Tezosar 500 SC in the 10RD dose, where its value increased. The EP value of fungi significantly increased in the soil amended with Sulcogan 300 SC in the RD dose and also with Tezosar 500 SC in the RD and 10RD doses, compared to the control soil. The greatest biodiversity was demonstrated for the actinobacteria (EP values from 0.852 to 0.899), followed by organotrophic bacteria (EP values from 0.789 to 0.848), and the least for fungi (EP values from 0.538 to 0.763).

**Table 3:** Effect of herbicides on ecophysiological diversity index of microorganisms (EP).

Object	Org	Act	Fun
C	0.790 ± 0.019 ^c^	0.867 ± 0.003 ^bc^	0.538 ± 0.020 ^d^
SulRD	0.822 ± 0.0131 ^ab^	0.879 ± 0.001 ^b^	0.632 ± 0.005 ^cd^
Sul10RD	0.846 ± 0.003 ^a^	0.874 ± 0.003 ^b^	0.667 ± 0.009 ^bc^
TezRD	0.789 ± 0.010 ^c^	0.882 ± 0.005 ^ab^	0.705 ± 0.031 ^ab^
Tez10RD	0.848 ± 0.004 ^a^	0.889 ± 0.002 ^a^	0.763 ± 0.042 ^a^
SucRD	0.823 ± 0.013 ^ab^	0.870 ± 0.013 ^b^	0.585 ± 0.029 ^de^
Suc10RD	0.814 ± 0.001 ^bc^	0.852 ± 0.010 ^c^	0.580 ± 0.029 ^de^

C—control soil; Sul—Sulcogan 300 SC; Tez—Tezosar 500 SC; Suc—Sulcotrek 500 SC; RD—dose recommended by the manufacturer; 10RD—dose 10-fold higher than recommended by the manufacturer; Org—organotrophic bacteria; Act—actinobacteria; Fun—fungi; ±—standard deviation. Homogeneous groups denoted by letters (^a–e^) were calculated separately for each group of microorganisms.

**Figure 1:**
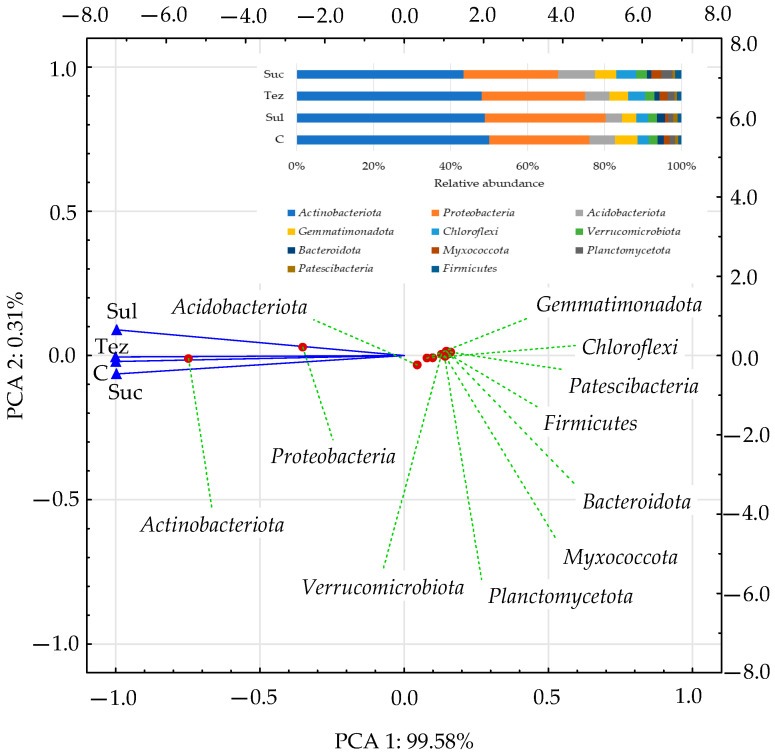
Dominant phylum of bacteria (OTU ≥ 1%) in soil presented as PCA. C—control soil; Sul—Sulcogan 300 SC; Tez—Tezosar 500 SC; Suc—Sulcotrek 500 SC.

The data presented in Figure 1 show that the soil was most intensely colonized by bacteria belonging to the phylum, *Actinobacteriota*, (accounting for 43.37% to 50.01%) and *Proteobacteria* (accounting for 24.64% to 31.43%), with the population number of *Actinobacteriota* being significantly higher than that of *Proteobacteria*. The abundance of *Actinobacteriota* and *Proteobacteria* in the herbicide-treated soils was significantly greater than in the control soil, with the highest number of these bacteria observed in the soil treated with the Sulcogan 300 SC herbicide. The bacteria belonging to the remaining phyla were found in the soil in much smaller numbers. In PCA, they formed a homogeneous group composed of *Acidobacteriota*, *Gemmatimonadota*, *Chloroflexi*, *Bacteroidota*, *Firmicutes*, *Myxococcota*, *Patescibacteria*, *Planctomycota*, and *Verrucomicrobiota* bacteria.

**Figure 2:**
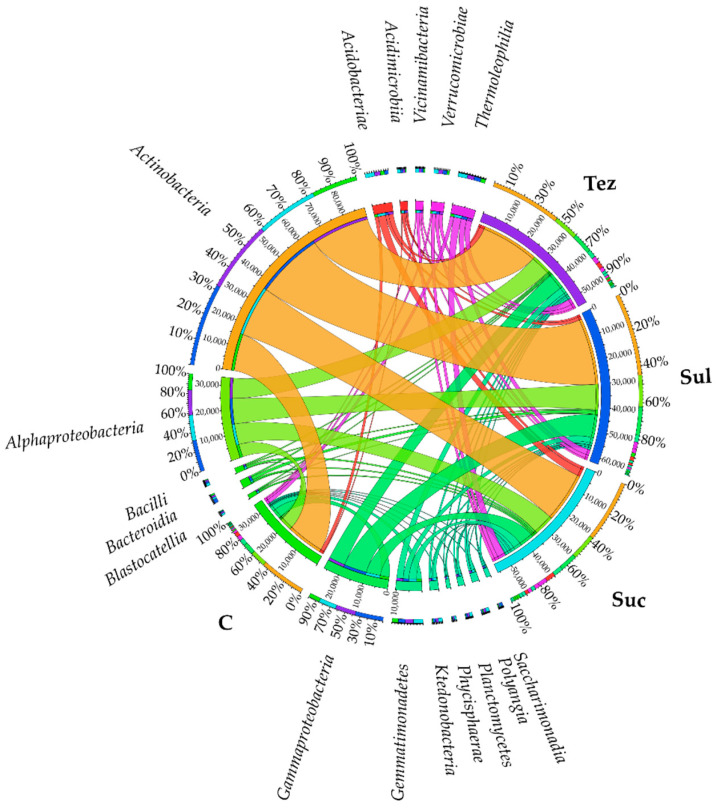
Dominant class of bacteria (OTU ≥ 1%) in soil. C—control soil; Sul—Sulcogan 300 SC; Tez—Tezosar 500 SC; Suc—Sulcotrek 500 SC.

Both the control and the herbicide-treated soil were the most heavily populated by *Actinobacteria*, belonging to the phylum, *Actinobacteriota*. Their OTU number was the highest in the soil treated with Sulcogan 300 SC herbicide (28,138 OTU) and the lowest in the control soil (15,521 OTU). In these soil types, there were also numerous *Alphaproteobacteria* and *Gammaproteobacteria*, bacteria belonging to the phylum, *Proteobacteria*. Their OTU number was highest in soils with the addition of Sulcogan 300 SC and reached 10,706 OTU and 9576 OTU, respectively (Figure 2).

**Figure 3:**
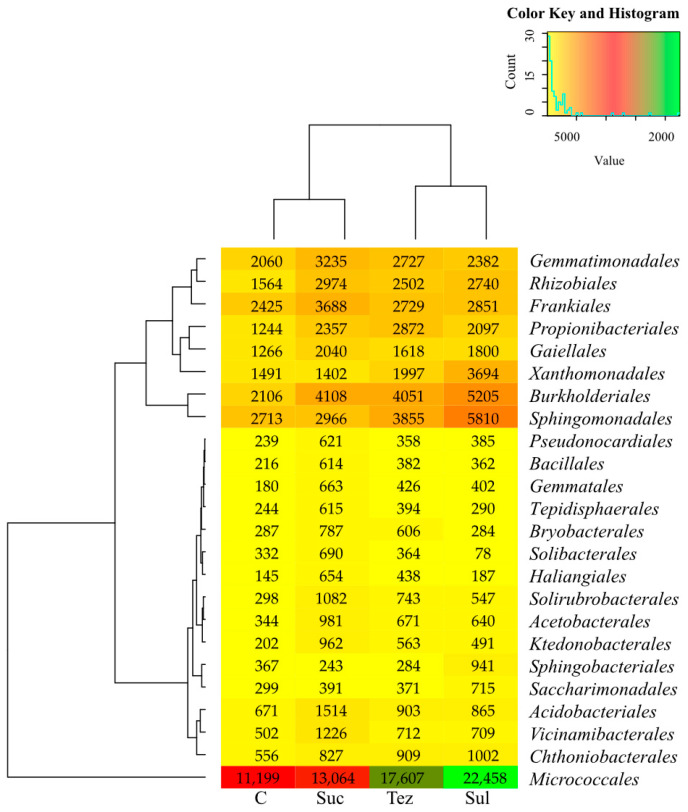
Dominant order of bacteria (OTU ≥ 1%) in soil. C—control soil; Sul—Sulcogan 300 SC; Tez—Tezosar 500 SC; Suc—Sulcotrek 500 SC.

Dominant bacterial orders are shown in Figure 3. The *Micrococcales* order, representing the *Actinobacteria* class, prevailed in all analyzed soil samples. The number of these bacteria ranged from 11,199 OTU (C soil) to 22,458 OTU (soil treated with Sul). In addition, the soil was densely colonized by bacteria belonging to the orders, *Frankiales*, *Propionibacteriales* and *Gaiellales* (class *Actinobacteria*), *Sphingomonadales* and *Rhizobiales* (class *Alphaproteobacteria*), *Burkholderiales* (class *Betaproteobacteria*), *Xanthomonadales* (class *Gammaproteobacteria*), and *Gemmatimonadales* (class *Gmmatimonadetes*).

**Figure 4:**
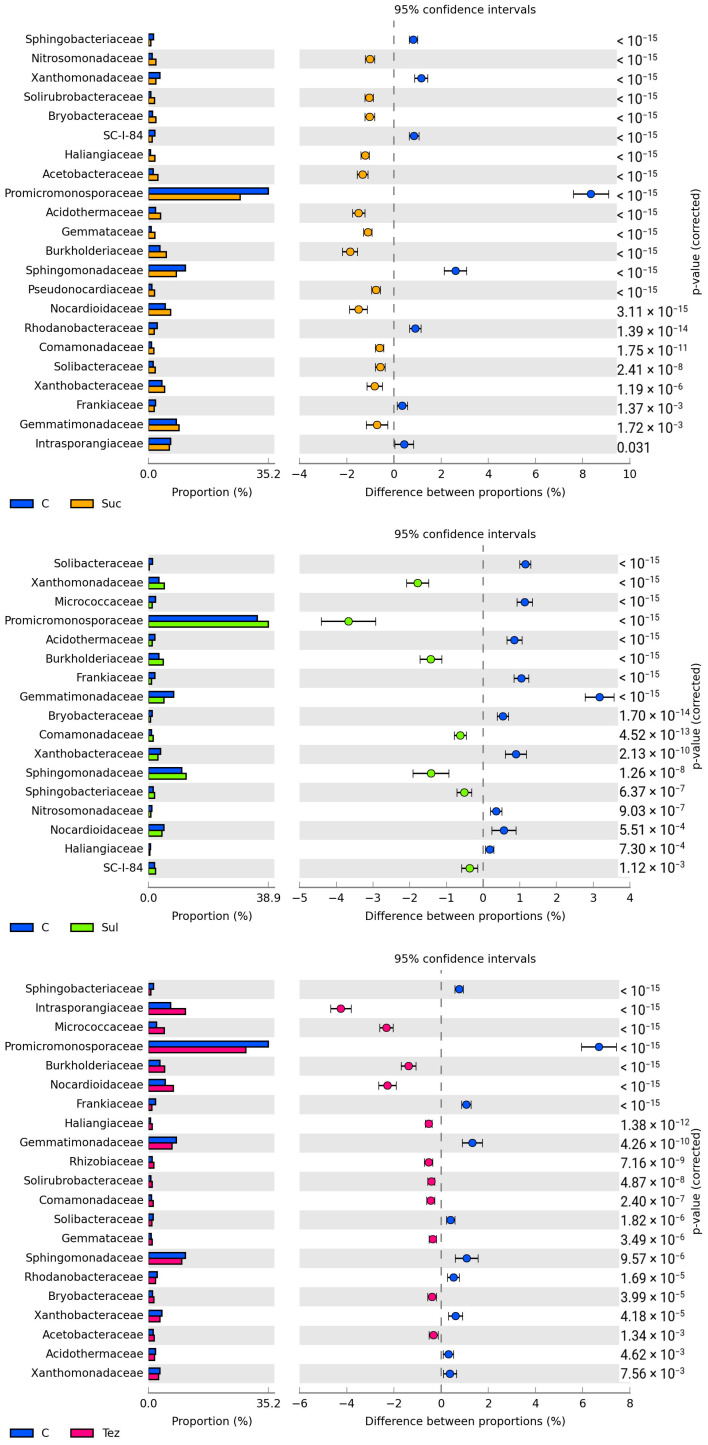
Dominant family of bacteria (OTU ≥ 1%) in soil. C—control soil; Sul—Sulcogan 300 SC; Tez—Tezosar 500 SC; Suc—Sulcotrek 500 SC.

In all analyzed soil samples, the *Promicromonosporaceae* family of bacteria was found to predominate (accounting for 26.83% to 38.85%). In the soil treated with the Sulcogan 300 SC herbicide, their number increased by 3.67% compared to the control soil, whereas in the soil with the addition of Tezosar 500 SC and Sulcotrek 500 SC, their number decreased by 6.69% and 8.35%, respectively. The soil was also heavily populated by bacteria belonging to the *Sphingomonadaceae* family, with their abundance increasing under the influence of the Sulcogan 300 SC herbicide by 1.42% compared to the control soil, and decreasing by 1.08% and 2.61% in the soil treated with the Tezosar 500 SC and Sulcotrek 500 SC herbicides, respectively. The Sulcogan 300 SC and Tezosar 500 SC herbicides inhibited the growth of *Gemmatimonadaceae*, *Acidothermaceae*, and *Solibacteraceae*, while Tezosar 500 SC and Sulcotrek 500 SC stimulated the multiplication of *Nocardioidaceae*, *Acetobacteraceae*, *Solirubrobacteraceae*, and *Haliangiaceae* bacteria. In addition, all the tested herbicides contributed to the increasing counts of *Burkholderiaceae*, *Comamonadaceae*, and *Gemmataceae* bacteria and the decreasing counts of *Frankiaceae* (Figure 4).

**Figure 5:**
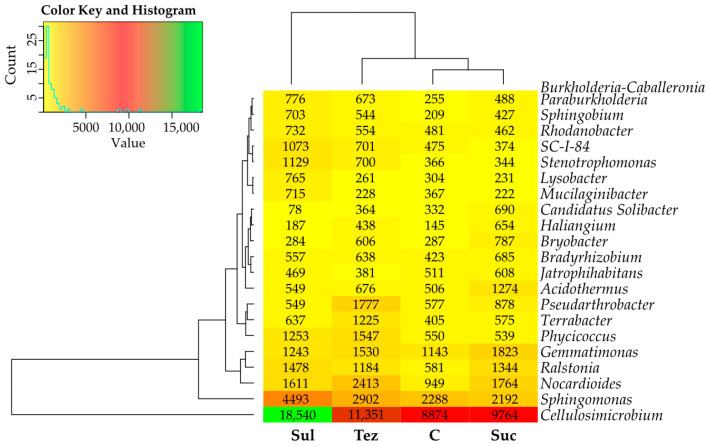
Dominant genus of bacteria (OTU ≥ 1%) in soil. C—control soil; Sul—Sulcogan 300 SC; Tez—Tezosar 500 SC; Suc—Sulcotrek 500 SC.

The soil was most colonized by bacteria of the phylum, *Cellulosimicrobium*, with their numbers ranging from 8874 OTU (C soil C) to 18,540 OTU (soil treated with Sul). The soil was also heavily populated by the *Sphingomonas* genus bacteria, the high population number of which (reaching 4493 OTU) was observed in the soil treated with Sulcogan 300 SC. The application of Tezosar 500 SC and Sulcotrek 500 SC to the soil reduced the counts of *Mucilaginibacter* and *Lysobacter* genus bacteria, and increased that of the *Pseudarthrobacter* and *Candidatus_Solibacter* bacteria. In turn, all tested herbicides increased the abundance of the *Acidothermus*, *Gemmatimonas*, *Nocardioides*, *Ralstonia, Bradyrhizobium*, *Terrabacter*, *Burkholderia-Caballeronia-Paraburkholderia*, *Sphingobium,* and *Haliangium* genus bacteria (Figure 5).

**Figure 6:**
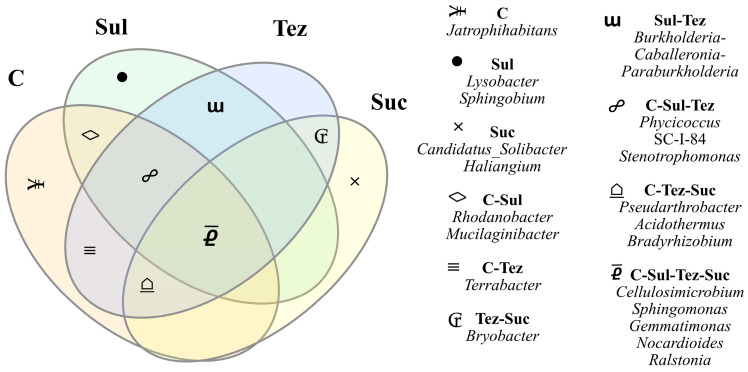
Unique and common genera of bacteria shown in the form of a Venn diagram. C—control soil; Sul—Sulcogan 300 SC; Tez—Tezosar 500 SC; Suc—Sulcotrek 500 SC.

All common and unique bacterial genera were selected based on the Venn diagram shown in Figure 6. All soil samples contained bacteria of the *Cellulosimicrobium*, *Sphingomonas*, *Gemmatimonas*, *Nocardioides*, and *Ralstonia* genera. Unique bacteria belonging to the *Lysobacter* and *Sphingobium* genera appeared in the Sulcogan 300 SC-treated soil, and in the *Candidatus_Solibacter* and *Haliangium* genus bacteria, in the soil treated with the Sulcotrek 500 SC herbicide.

**Figure 7:**
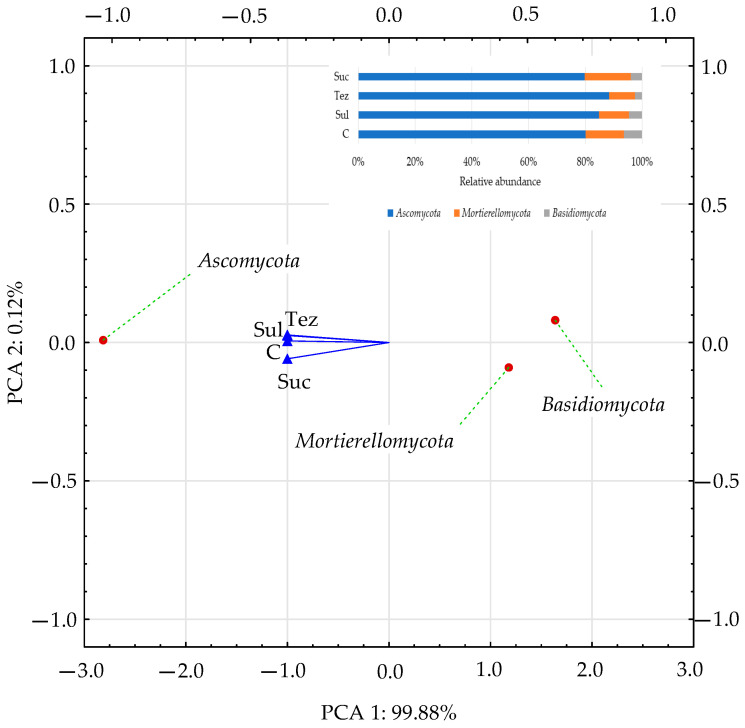
Dominant phylum of fungi (OTU ≥ 1%) in soil presented as PCA. C—control soil; Sul—Sulcogan 300 SC; Tez—Tezosar 500 SC; Suc—Sulcotrek 500 SC.

The dominant phylum of fungi turned out to be *Ascomycota* (accounting for 79.77% to 88.40%), followed by *Mortiellomycota* (accounting for 9.05% to 16.27%) and *Basidiomycota* (accounting for 2.55% to 6.36%). *Ascomycota* were the most abundant in the soil treated with Tezosar 500 SC, whereas *Mortiellomycota* was the most common in the soil treated with Sulcotrek 500 SC. The relative abundance of fungi belonging to the phylum, *Basidiomycota*, decreased significantly in the herbicide-treated soil compared to the control soil (Figure 7). 

**Figure 8:**
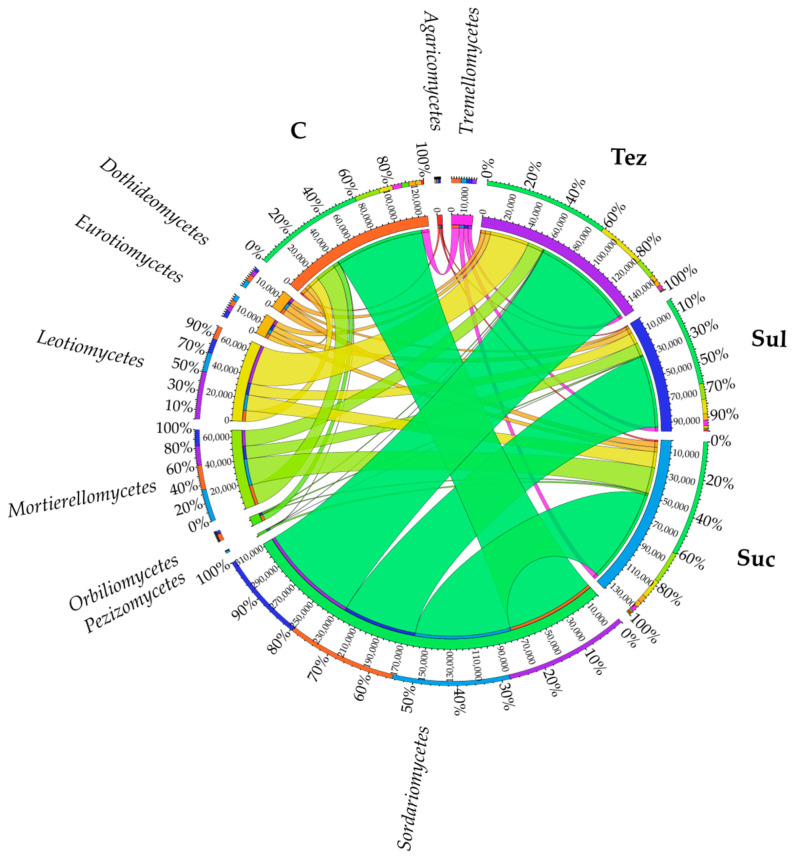
Dominant class of fungi (OTU ≥ 1%) in soil. C—control soil; Sul—Sulcogan 300 SC; Tez—Tezosar 500 SC; Suc—Sulcotrek 500 SC.

At the class level, *Sordariomycetes* fungi prevailed in the soil; however, their OTU number decreased by 18.18% in the soil treated with Sulcogan 300 SC compared to the control soil, and increased by 13.26% and 6.19% in the soil treated with Tezosar 500 SC and Sulcotrek 500 SC, respectively. There were also *Mortierellomycetes* class fungi (their numbers ranged from 11,272 OTU to 23,045 OTU) and *Leotiomycetes* class fungi (their numbers ranged from 9522 OTU to 33,936 OTU) in the soil. The remaining fungal classes at OTU ≥ 1% (*Tremellomycetes*, *Orbiliomycetes*, *Dothideomycetes*, *Eurotiomycetes*, *Agaricomycetes*, and *Pezizimycetes*) occurred in the soil in slightly smaller numbers (Figure 8).

**Figure 9:**
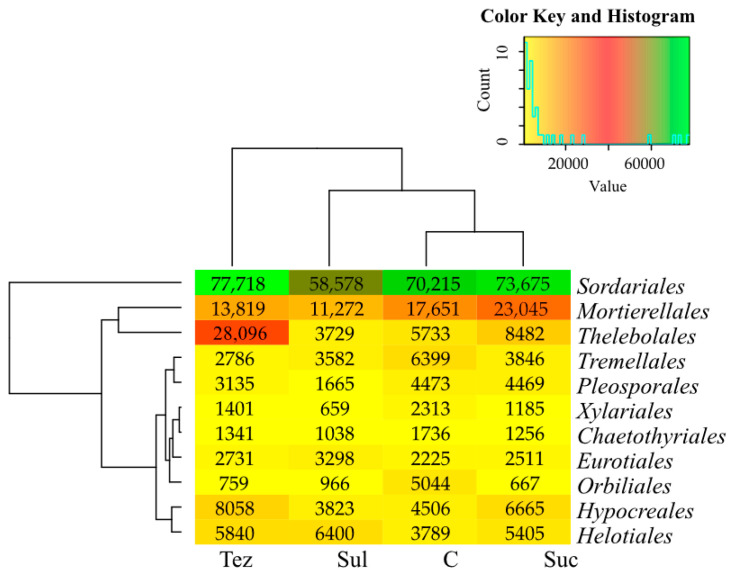
Dominant order of fungi (OTU ≥ 1%) in soil. C—control soil; Sul—Sulcogan 300 SC; Tez—Tezosar 500 SC; Suc—Sulcotrek 500 SC.

The most abundant fungi found in the analyzed soil samples were those belonging to the order, *Sordariales* (Figure 9), with their numbers ranging from 58,578 OTU (Sul-treated soil) to 77,718 OTU (Tez-treated soil). In addition, all soil types were heavily colonized by fungi from the order, *Mortierellales.* (Their numbers ranged from 11,272 OTU to 23,045 out.) A great abundance of the *Thelebolales* order fungi was also found in the soil treated with the Tezosar 500 SC herbicide (28,096 OTU).

**Figure 10:**
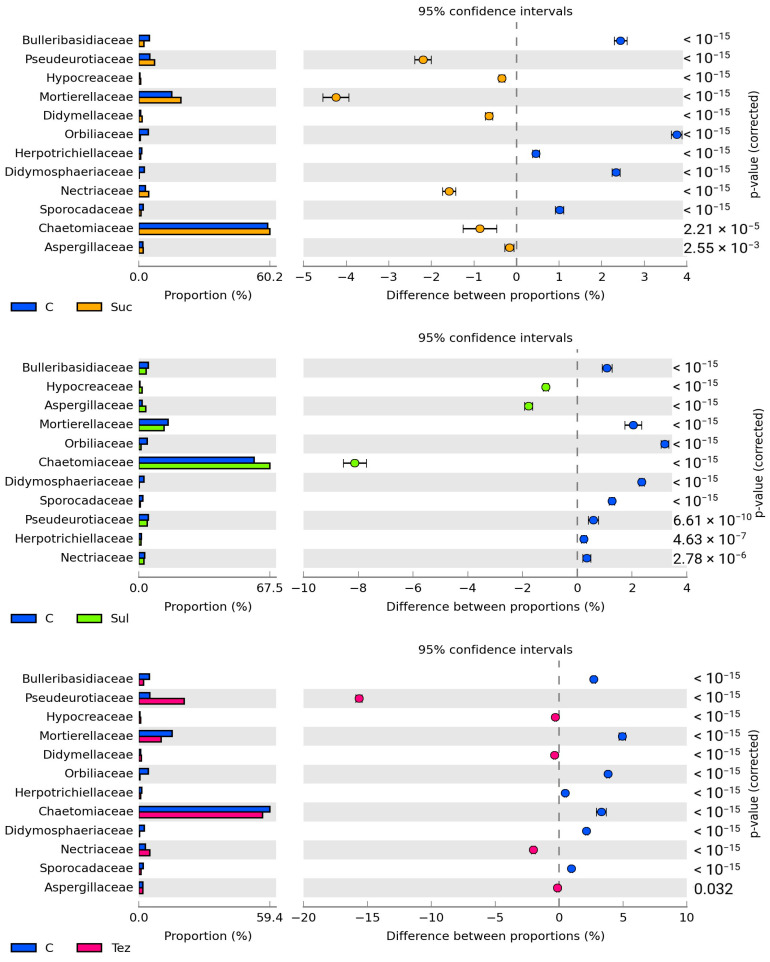
Dominant family of fungi (OTU ≥ 1%) in soil. C—control soil; Sul—Sulcogan 300 SC; Tez—Tezosar 500 SC; Suc—Sulcotrek 500 SC.

The application of herbicides into the soil resulted in changes in the structure of fungal communities at the family level (Figure 10). The soil was most colonized by the fungi from the *Chaetomiaceae* family. (Their numbers ranged from 67.49% to 56.05%.) Their abundance increased by 8.13% in the soil treated with Sulcogan 300 SC and decreased by 3.31% in the soil treated with Tezosar 500 SC. In the herbicide-treated soil, there was also a significant increase in the number of *Pseudeurotiaceae* fungi (by 15.67%) compared to the control soil. The abundance of *Mortierellaceae* fungi increased by 4.24% upon soil treatment with Sulcotrek 500 SC, and decreased in the soils treated with Sulcogan 300 SC and Tezosar 500 SC by 2.05% and 4.96%, respectively. All the herbicides tested reduced the abundance of the *Didymosphaeriaceae*, *Orbiliaceae*, *Sporocadaceae*, and *Bulleribasidiaceae* family fungi.

**Figure 11:**
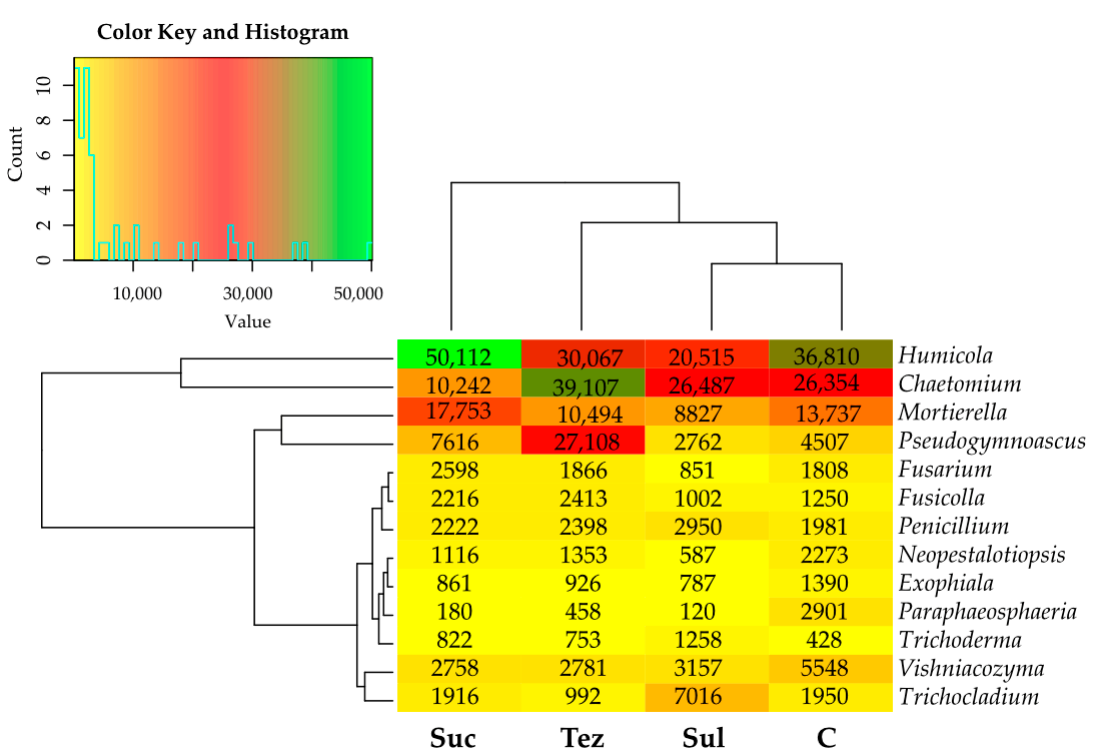
Dominant genus of fungi (OTU ≥ 1%) in soil. C—control soil; Sul—Sulcogan 300 SC; Tez—Tezosar 500 SC; Suc—Sulcotrek 500 SC.

The soil samples were most heavily populated by the fungi from the *Humicola* and *Chaetomium* genera, belonging to the *Chaetomiaceae* family, and slightly less by the *Morierella* genus fungi, belonging to the *Mortierellaceae* family (Figure 11). The greatest abundances of the fungi from the *Humicola* and *Morierella* genera were recorded in the soil treated with Sulcotrek 500 SC (their numbers reached was 50,112 OTU and 17,153 OTU, respectively) and that of *Chaetomium* fungi in the soil amended with Tezosar 500 SC (39,107 OTU). The soil treated with Tezosar 500 SC was also densely colonized by the *Pseudogymnoascus* genus fungi (27,108 OTU).

**Figure 12:**
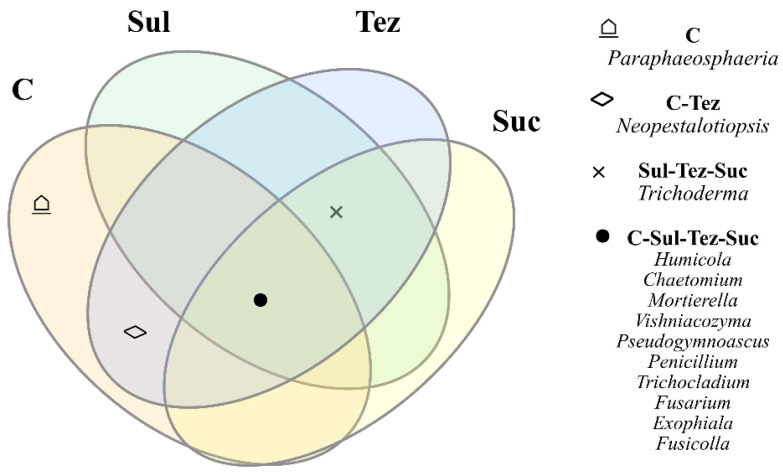
Unique and common genera of fungi shown in the form of a Venn diagram. C—control soil; Sul—Sulcogan 300 SC; Tez—Tezosar 500 SC; Suc—Sulcotrek 500 SC.

In both control and herbicide-treated soils, 10 common genera of fungi have been identified, including *Humicola*, *Chaetomium*, *Mortierella*, *Vishniacozyma*, *Pseudogymnoascus*, *Penicillium*, *Trichocladium*, *Fusarium*, *Exophiala*, and *Fusicolla*. In addition, the soils containing the Sulcogan 300 SC, Tezosar 500 SC, and Sulcotrek 500 SC herbicides were colonized by the fungi of the genus *Trichoderma* (Figure 12).

### 2.2. Response of Soil Enzymes to Herbicides

The response of enzymes to the herbicides applied to the soil varied and depended on herbicide type and dose (Table S2). All herbicides applied to soil at the dose recommended by the manufacturer significantly inhibited the activity of dehydrogenases and urease, while they stimulated the activity of arylsulfatase and acid phosphatase relative to the control soil. The formulations applied at this dose did not cause significant changes in *β*-glucosidase activity. Regardless of the dose, all herbicides inhibited urease activity but stimulated the activities of acid phosphatase and arylsulfatase. The Tezosar 500 SC herbicide applied in the 10RD dose stimulated the activity of *β*-glucosidase. In addition, inhibited activities were observed in the case of dehydrogenases—in SulRD, Sul10RD, TezRD and SucRD soil samples; catalase—in Sul10RD and Suc10RD soil samples; and alkaline phosphatase—in the Sul10RD soil sample. In contrast, enhanced activity was observed in the case of alkaline phosphatase in TezRD, Tez10RD, and SucRD soil samples.

The IF values show that the herbicides had a significant effect on soil enzymatic activity (Table 4). 

Dehydrogenases and urease turned out to be the most sensitive to the tested preparations, as evidenced by the negative values of their IFs. The IF values determined for dehydrogenases ranged from −0.079 to −0.155, whereas those computed for urease ranged from −0.233 to −0.510. The most negative effect on urease activity was caused by Sulcotrek 500 SC and on the dehydrogenases, by Sulcogan 300 SC. All IF values computed for acid phosphatase and arylsulfatase were positive, which may indicate a stimulating effect of the herbicides on their activity. The IF values computed for acid phosphatase were in the range of 0.078 to 0.298 and those computed for arylsulfatase, in the range of 0.104 to 0.486. The most beneficial effect on the activity of acid phosphatase was also exerted by Tezosar 500 SC, while on the activity of arylsulfatase, it was by Sulcogan 300 SC. It was observed that catalase responded negatively to Sulcogan 300 SC and Sulcotrek 500 SC applied to the soil in the 10RD dose (IF = −0.049 and IF = −0.051, respectively); alkaline phosphatase, to Sulcogan 300 SC added in the RD dose (IF = −0.044), and 10RD dose (IF = −0.220) and to Sulcotrek 500 SC applied in the RD dose (IF = −0.073); and *β*-glucosidase, to Sulcogan 300 SC introduced in the soil in the RD (IF = −0.070) and Tezosar 500 SC applied in the 10RD dose (IF = −0.070).

### 2.3. Response of Maize (Zea mays L.) to Herbicides

**Figure 13:**
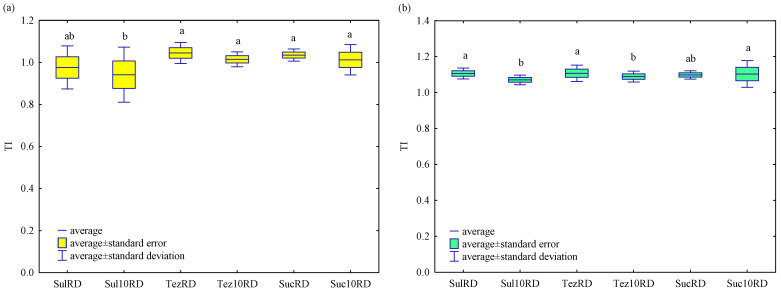
Tolerance index (TI) of *Zea mays* L. to herbicides based on (**a**) plant yield and (**b**) length of aboveground plant parts. Sul—Sulcogan 300 SC; Tez—Tezosar 500 SC; Suc—Sulcotrek 500 SC; RD—dose recommended by the manufacturer; 10RD— dose 10-fold higher than recommended by the manufacturer. Homogeneous groups are indicated by letters (^a,b^).

**Figure 14:**
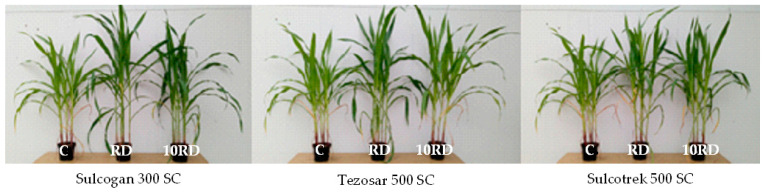
*Zea mays* L. on day of harvest at BBCH 39 stage (60th day of vegetation). C—control soil; RD—dose recommended by the manufacturer; 10RD—dose 10-fold higher than recommended by the manufacturer.

The herbicides tested did not adversely affect the growth and development of *Zea mays* L. (Figure 13, Figure 14 and Figure S1). The dose recommended by the manufacturer of the herbicides, Tezosar 500 SC and Sulcotrek 500 SC, caused a statistically significant increase in the yield of the dry weight of the aboveground parts of maize compared to the control sample. The length of aboveground parts of plants increased after application of the dose recommended by the manufacturer of the herbicides, Sulcogan 300 SC and Tezosar 500 SC. Sulcogan 300 SC, caused no significant changes in the yield of plants, whereas the remaining herbicides stimulated the growth and development of maize. These observations were also confirmed by the TI values exceeding 1 (Figure 13a). Given the length of the aboveground parts of the plants, it can be concluded that maize was tolerant to the herbicides. The TI values were in the range of 1.071 to 1.108 (Figure 13b).

**Figure 15:**
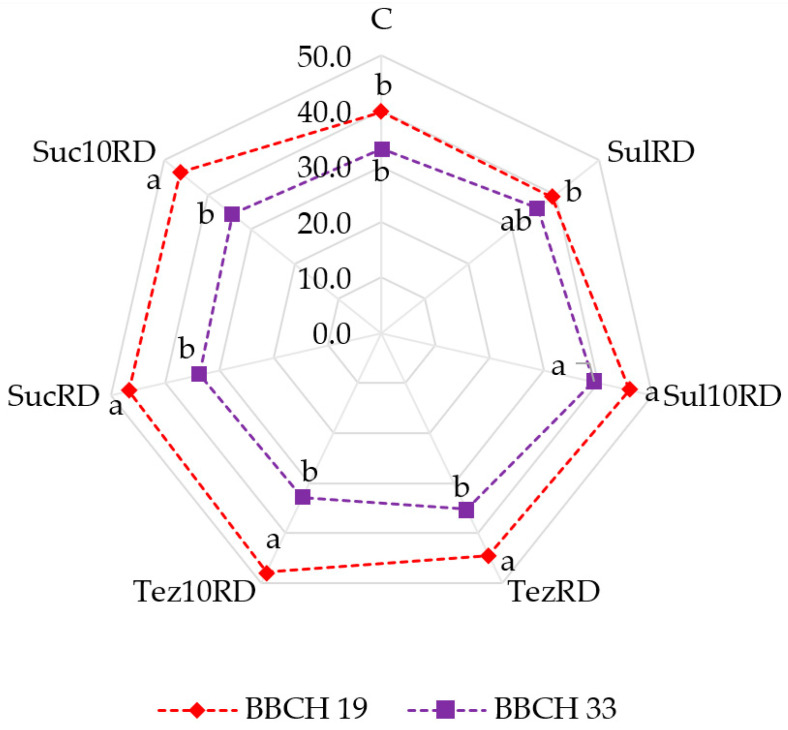
Effect of herbicides on leaf greenness index (SPAD) of *Zea mays* L. C—control soil; Sul—Sulcogan 300 SC; Tez—Tezosar 500 SC; Suc—Sulcotrek 500 SC; RD—dose recommended by the manufacturer; 10RD—dose 10-fold higher than recommended by the manufacturer; BBCH 19—9th leaf stage; BBCH 33—stage 3 knees. Homogeneous groups denoted by letters (^a,b^) were calculated separately for each developmental stage of maize.

Soil treatment with the tested herbicides also modified the values of the leaf greenness index *Zea mays* L. (Figure 15). When the maize was at the BBCH 19 development stage, the values of its SPAD index determined when grown in the herbicide-treated soil increased from 11.29% to 19.83% compared to the control sample. Only Sulcogan 300 SC applied in the dose recommended by the manufacturer did not cause any significant modifications to the leaf greenness index. At the BBCH 33 development stage of the maize, its value was lower than in the BBCH 19 stage. Sulcogan 300 SC applied in the 10RD dose increased the SPAD index compared to the control sample, while the other herbicides caused no significant changes in its values.

## 3. Discussion

### 3.1. Response of Soil Microorganisms to Herbicides

The preservation of the microbial diversity of soil is crucial to maintaining its appropriate quality and health. However, this balance may be disturbed by anthropogenic factors such as the excessive use of pesticides, which may cause perturbations in both the diversity and the structure of bacterial and fungal communities [30]. The present research has focused on the impact of three herbicides used to protect maize on the population numbers, diversity, and structure of bacterial and fungal communities in the soil. It has been found that soil treatment with herbicides yielded conditions most favorable to the development of organotrophic bacteria and slightly less favorable for actinobacteria. In the case of fungi, their growth was clearly inhibited, especially in the soil treated with the highest tested dose of the analyzed herbicides. Soil amendment with the herbicides also caused changes in the proportions between the r-strategists and K-strategists. These relationships prove that the organotrophic bacteria and actinobacteria have a greater contribution in the degradation of the analyzed herbicides than fungi. In addition, the soil may have been more densely colonized by bacteria and actinobacteria, which used the available active substances of these preparations as a source of nutrients, making their development more intense than that of fungi [31,32]. Bacterial populations that survived in the herbicide-contaminated soil could also develop intensively by using the dead cells of other microorganisms as an alternative source of carbon [33]. In addition, the resistance of organotrophic bacteria to the tested herbicides may have been associated with the presence in their cells of enzymes that control the polymorphic catalase responsible for oxidative stress, as well as structural changes in lipid membranes, which act as a selective barrier against herbicides [34]. In turn, the inhibition of fungal growth by herbicides may have been caused by oxidative stress resulting from the production of free radicals, leading to damage to the cell’s structure [35]. Sariwati et al. [36] have reported that fungal hyphae serve as transport vectors for bacteria, thus facilitating the spread of species capable of effectively degrading pesticides. Such a relationship between bacteria and fungi could also exist in the present study.

Dong et al. [37] have emphasized that, in order to survive in herbicide-contaminated soils, microorganisms must be capable of the horizontal transfer of genes encoding enzymes, such as monooxygenases, which are involved in the degradation of these chemicals. Such genes have been found in, among other things, bacteria of the *Pseudomonas* and *Arthrobacter* genera, which are considered viable herbicide degraders [34]. In turn, the *Arthrobacter aurescens* strain of TC1 has been deemed an effective bacterium degrading terbuthylazine. The catabolism of terbuthylazine by these bacteria occurs via the hydrolytic displacement of the chlorine and amino substituents of the s-triazine ring. This process is aided by TrzN enzymes catalyzing the initial stage of dechlorination, which produces the hydroxy-s-triazine metabolite as well as AtzB and AtzC, which catalyze the sequential deamination of N-alkyl side amines [38]. The conversion of terbuthylazine in soil is very important for the environment, as the resulting metabolites are less toxic than the initial compound itself [6,38]. According to Tonello et al. [6], the most active in the terbuthylazine transformation in the soil are bacteria belonging to the following classes: *Betaproteobacteria* (represented by *Advenella incenata* and *Janthinobacterium lividum*), *Gammaproteobacteria* (represented by the *Pseudomonas* sp. strain MHP41, and *Arthrobacter* sp.), and *Actinobacteria* (represented by *Rhodococcus wratislaviensis* FPA 1). Petric et al. [39] and Merlin et al. [40] reported that soils contaminated with triketone-group herbicides, such as sulcotrione, were the most abundant in bacteria belonging to the *Actinobacteriota* and *Bacteroidota* phyla, while Romdhane et al. [41] showed *Proteobacteria* and *Acidobacteriota* to be the prevailing phyla. The results obtained in the present research confirm findings from previous studies. They show that the analyzed soil was most heavily populated by bacteria belonging to the *Actinobacteriota* phylum, with their numbers being significantly reduced under the influence of the Sulcotrek 500 SC herbicide. The response of these bacteria to the herbicides tested may be due to the greater stability of the mixture of its active substances and thus to their lesser availability to bacteria capable of degrading these substances [42]. Another phylum abundant in the soil was *Proteobacteria*, whose relative abundance increased significantly after soil treatment with the Sulcogan 300 SC herbicide. In order to survive under adverse conditions, these bacteria could produce new enzymes and undergo a number of genetic mutations, which allowed them to adapt to new conditions and modified environment [21]. Regardless of herbicide type, the soil analyzed was most heavily populated by the bacteria of the *Cellulosimicrobium* genus, which are probably capable of degrading these preparations. Additionally, bacteria from of the *Sphingomonas* and *Gemmatimonas* genera may exhibit high resistance to the tested herbicides, because their numbers were significantly higher in the soil treated with these preparations than in the control soil. This may indicate their adaptation to the new conditions changing in the soil environment under the influence of herbicides. According to Tonello et al. [6], the most active fungi in the degradation of herbicides from the triketone group are *Aspergillus oryzae* and *Penicillium brevicompactum*, belonging to the class, *Eurotiomycetes*, and *Lentimula edode*, belonging to the class, *Agaricomycetes*. In the present research, the soil was mainly colonized by the fungi from the *Humicola* and *Chaetomium* genera, belonging to the *Sordariomycetes* class and by *Mortierella*, belonging to the *Mortierellomycetes* class. The response of the above-mentioned fungal genera varied depending on the herbicide used. The development of the *Humicola* genus fungi was inhibited by Sulcogan 500 SC and Tezosar 500 SC, whereas their growth was stimulated by Sulcotrek 500 SC. The soil amendment with Tezosar 500 SC had a positive effect on the development of *Chaetomim*, and soil treatment with Sulcotrek 500 SC, on the development of *Mortierella* genus fungi. An increase observed in the number of fungi under the influence of herbicides may indicate their greater resistance to these preparations and their degradation potential. The biodegradation of herbicides by fungi is based on an oxidative-hydrolytic mechanism, during which they modify the molecule by sequentially removing the aromatic ring substituent, with dealkylation as the initial stage of herbicide degradation [43]. It has also been observed in this study that the number of fungi of the *Mortierella* genus was reduced under the influence of Sulcogan 300 SC and Tezosar 500 SC, while the number of the *Chaetomium* genus fungi, after Sulcotrek 500 SC application. The inhibition of fungal growth may have been due to protein overexpression associated with increased energy demand, changes in the cell wall and cytoskeleton, as well as a response to oxidative stress induced by herbicides [43].

### 3.2. Response of Soil Enzymes to Herbicides

The use of herbicides may disturb not only the populations and microbial diversity of the soil, but also its enzymatic activity. The activity of certain enzymes can be inhibited by herbicides applied to the soil, while that of others may be stimulated. These changes may occur depending on the herbicide type and dose, as well as the duration of the deposition of these xenobiotics in the soil [44]. According to Roman et al. [3], the activity of enzymes in soil is correlated with the dynamics of organic matter degradation and changes in biogeochemical cycles, which makes their response to stress factors so rapid. The conducted research shows that the tested herbicides had an inactivating effect on dehydrogenases and urease. The reason behind the inhibition of these soil enzymes was the rapid stress induced by the application of herbicides to the soil, leading to damage to the cells of microorganisms sensitive to these xenobiotics, which ultimately suppressed the secretion of enzymes by these microorganisms. This study also demonstrated a stimulating effect of herbicides on acid phosphatase and arylsulfatase activities, which may have been due to the high resistance of these enzymes, that may have been involved in the degradation of herbicides, to adverse environmental conditions [45]. As Kumar et al. [20], and Bhatt et al. [22] claim, pesticide degradation during hydrolysis is aided by many enzymes like hydrolases, esterases, phosphatases, phosphotriesterases, and carboxylesterases. Degrading enzymes are encoded by the expression of the plasmid gene and chromosome gene in bacteria, due to which they often exhibit greater resistance to pesticides than the microbial cells themselves [45]. The enhanced activity of these soil enzymes can also be explained by the increased abundance of the microorganisms, which, in their presence, more intensively degraded herbicides, using them as a source of carbon [46].

### 3.3. Response of Maize (Zea mays L.) to Herbicides

Maize is one of the most important crops in the world, and its growth may be inhibited by competing weeds. Its proper yielding may be ensured, foremost, by the use of herbicides that effectively eliminate weeds [47,48]. According to the research conducted, the response of maize to the herbicides applied varied depending on their type and dose. Tezosar 500 SC and Sulcotrek 500 SC increased the yield of dry matter of the plants, while Sulcogan 300 SC added to the soil did not cause statistically significant changes. This may be due to the stress caused by the herbicide application, which could lead to mass generation of reactive oxygen species and disturbances in the activity of antioxidant enzymes, which ultimately cause damage to plant cells, e.g., in the lipid membrane, caused by reactive oxygen species [49]. The herbicide, Sulcogan 300 SC, did not have a significant effect on the yield of dry matter of aboveground parts of maize in relation to the control soil (without the addition of herbicides), as the active ingredient of the herbicide may have been inactivated by soil microorganisms, so that there was a reduction in its absorption and penetration into plant cells [50,51]. Such a relationship may have led to a reduction in the effects of the herbicide on the corn, so that the yield of the dry weight of the aboveground parts of the plants did not change statistically significantly compared to the control soil under the influence of the tested preparation. In addition, this herbicide contains sulcotrione, known to inhibit p-hydroxyphenylpyruvate dioxygenase, which is responsible for tyrosine transformation. The impairment of this process impedes the synthesis of carotenoids, which leads to physiological changes in the plant and then to its death [52,53]. In turn, the increase in maize yield may be due to the effective removal of weeds in maize stands by the Tezosar 500 SC and Sulcotrek 500 SC herbicides, which improve plant condition [47]. These herbicides contain terbuthylazine, which is absorbed mainly through the roots of the weeds and to a lesser extent, through their leaves. Terbuthylazine is transported through the xylem to the apical meristems and leaves, causing, in the first place, leaf chloroses visible in the inter-venous spaces as well as on the edges and tops, followed by necroses, growth inhibition, drying and dying of weeds. This substance disturbs the course of photosynthesis, chlorophyll decay and cell membrane function, leading to weed death [54]. Additionally, Alptekin et al. [55] confirmed that when it is applied to the soil, it reduces the number of weeds, thus increasing the quality and quantity of maize crops. In the present study, maize responded positively to the effects of the tested herbicides, as evidenced by the obtained values of its tolerance index (TI) to these preparations. Yu et al. [49] reported a strong phytotoxic effect of terbuthylazine and atrazine (6 mg kg^−1^ dose) on maize, indicated by a decrease in the dry weight of plants, the length of their shoots and roots, and chlorophyll content. The reduction in chlorophyll content in the plant by herbicides may be caused by blocking the transport of electrons in the photosynthesis process. However, in the present study, the herbicides did not impair the photosynthesis, as we noted their positive effect on the greenness index of the maize leaves, especially at the BBCH 19 growth stage. Such a positive response of the maize to the herbicides applied may be due to the secretion of antioxidant enzymes by the plants, i.e., peroxidase or glutathione s-transferase, which are some of the most important metabolic enzymes in the plant responsible for the transformation of pesticides [49,56].

Microorganisms have a great impact on the disappearance of herbicides in the soil, as they degrade organic compounds present in the environment they inhabit as a result of their metabolic properties. Their ability to metabolize these xenobiotics is related to the activity of structural enzymes and induced enzymes, which are produced by microorganism cells. This response of microorganisms to herbicides present in the soil is related to their adaptive mechanism, which allows them to survive in changing environmental conditions. In addition, microorganisms use herbicides as a source of carbon and energy during the decomposition of herbicides, which makes them multiply intensively [57]. Such abilities in our study could be characterized by *Cellulosimicrobium* and *Nocardioides* bacteria belonging to the phylum, *Actinomycetota*; *Sphingomonas* and *Ralstonia*, belonging to the phylum, *Proteobacteria*; and *Gemmatimonas*, belonging to the phylum, *Gemmatimonadota*, which were identified in all soil samples. These are soil bacteria that promote plant growth and development and live in close association with plants. These microorganisms also show the ability to combat plant pathogens, induce systemic plant resistance to disease, and have antagonistic effects on other microorganisms [58]. Plants, in turn, modulate the rhizosphere microbiome [59] and selectively attract beneficial microorganisms [60] through signals transmitted through root secretions and the production of volatile compounds and phytohormones that help shape the plant rhizosphere microbiota. Cooperation between plants and microorganisms, as well as interactions between microbiota, are crucial in shaping plant health [61]. In our study, such reactions between herbicide-degrading microbes and corn may have occurred, which promoted the growth and development of the aboveground parts of this crop.

## 4. Materials and Methods

### 4.1. Experimental Design

The pot-vegetation experiment was conducted in a vegetation hall located at University of Warmia and Mazury in Olsztyn (Poland, Central Europe). Before setting up experiment, the soil was sifted through a sieve (mesh diameter of 5 mm), and then 3.4 kg of soil was weighed each and following amounts of micronutrients were added per pure component (mg kg^−1^ d.m. of soil): nitrogen (in the form of CO(NH_2_)_2_)—130 mg, phosphorus (in the form of KH_2_PO_4_)—100 mg, potassium (in the form of KH_2_PO_4_ + KCl)—130 mg, and magnesium (in the form of MgSO_4_ × 7H_2_O)—25 mg. The same fertilizer doses were applied in all combinations. In the respective sites, herbicides were added once in form of an aqueous emulsion at manufacturer’s recommended dose (RD) and 10-fold higher than the recommended manufacturer dose (10RD). The soil, along with micronutrients and herbicides, was thoroughly mixed and placed in polyethylene pots (capacity 3.7 dm^3^). The control soil was soil without addition of herbicides. On the same day, 8 maize grains each (variety LG 32.52) were sown into pots, and soil was moistened to 50% capillary water capacity. When the maize was at BBCH 10 stage (the first leaf emerging from the coleoptile), plants were aborted and 5 plants were left in each pot. During plant vegetation (60 days), soil moisture was monitored and maintained at a constant level (50% of capillary water capacity). The moisture content of soil was checked 3 times each day, and water losses were systematically supplemented with deionized water. According to BBCH scale (Biologische Bundesanstalt, Bundessortenamt and Chemical Scale), when *Zea mays* L. was at developmental stage BBCH 19 (9th leaf stage) and BBCH 33 (stage of 3 knees), the leaf greenness index in relative units of SPAD was determined using the Chlorophyll Meter SPAD 502 Plus Product Manual (Konica Minolta, Langenhagen, Germany). The measurements were made in 5 replicates. At maize stage BBCH 39 (stage of 9 or more knees), the length of aboveground parts of plants and dry weight of plants were determined (fresh weight samples of plants were dried for 5 days at 65 °C). Moreover, at same time, soil samples were collected for microbiological and enzymatic analyses. The experiment included seven combinations, and each combination was carried out in 5 repetitions. The experimental scheme was as follows:

1. Soil without addition of herbicides (control);
2. Soil with addition of herbicide Sulcogan 300 SC at a dose recommended by the manufacturer (0.150 mg kg^−1^ d.m. Soil per active substance—sulrd);
3. Soil with addition of Sulcogan 300 SC herbicide at a dose 10-fold higher than recommended by the manufacturer (1.50 mg kg^−1^ d.m. Soil per active substance—Sul10RD);
4. Soil with addition of herbicide Tezosar 500 SC at a dose recommended by the manufacturer (0.165 mg kg^−1^ d.m. Soil per active substance—tezrd);
5. Soil with addition of Tezosar 500 SC herbicide at a dose 10-fold higher than recommended by the manufacturer (1.65 mg kg^−1^ d.m. Soil per active substance—Tez10RD);
6. Soil with addition of herbicide Sulcotrek 500 SC at a dose recommended by the manufacturer (0.333 mg kg^−1^ d.m. Soil per active substance—sucrd);
7. Soil with addition of Sulcotrek 500 SC herbicide at a dose 10-fold higher than recommended by the manufacturer (3.33 mg kg^−1^ d.m. soil per active substance—Suc10RD).

### 4.2. Soil

The soil used in experiment came from Tomaszkowo (53.7161° N, 20.4167° E), near Olsztyn in north-eastern Poland (Central Europe). The soil was taken from the arable-humic layer at a depth of 0–20 cm. The soil material in terms of grain size was classified as sandy loam (sandy—63.61%, silt—32.68%, clay—3.71%), belonging to Eutric Cambisols [62]. The soil was characterized by the following properties: pH_KCl_—4.40; hydrolytic acidity—26.10 mmol^+^ kg^−1^; sum of exchangeable base cations—63.60 mmol^+^ kg^−1^; cation exchange capacity—89.70 mmol^+^ kg^−1^; alkaline cation saturation—70.90%; organic carbon content—10.0 g kg^−1^; total nitrogen content—0.83 g kg^−1^; C to N ratio—12.05; exchangeable cations content (mg kg^−1^): K^+^—168.0; Na^+^—10.0, Ca^2+^—11.50, Mg^2+^—82.10. Physicochemical analyses of soil were performed according to procedure described by Wyszkowska et al. [63].

The soil was characterized by the following number of microorganisms per cfu kg^−1^ d.m. soil: organotrophic bacteria—0.562 × 10^10^, actinomycetes—0.237 × 10^10^ and fungi—0.405 × 10^8^, and activity of soil enzymes in kg^−1^ d.m. soil h^−1^: dehydrogenases—8.958 µmol TFF, catalase—0.365 mol O_2_, alkaline phosphatase—0.499 mmol PNP, acid phosphatase—3.128 mmol PNP, *β*-glucosidase—0.163 mmol PNP, arylsulfatase—0.269 mmol PNP, and urease—0.601 mmol N-NH_4_.

### 4.3. Herbicides

Three herbicides were applied to the soil in the study: Sulcogan 300 SC (Sul), Tezosar 500 SC (Tez), and Sulcotrek 500 SC (Suc). The tested preparations are used to protect maize against monocotyledonous and dicotyledonous weeds.

Sulcogan 300 SC was manufactured by Nufarm Polska Sp. z o. o. (Warsaw, Poland), and it was registered in Poland in 2012. In its composition, it contains an active ingredient called sulcotrione (a compound of triketone group) in amount of 300 g dm^−3^. The dose recommended by manufacturer is 1.5 dm^3^ ha^−1^. It is a selective herbicide with systemic action, which comes in the form of a concentrate for dilution with water.

Tezosar 500 SC was produced by Ciech Sarzyna S.A. (Nowa Sarzyna, Poland), launched in Poland in 2018. The active ingredient of this preparation is terbuthylazine (a compound from triazine group), present in amount of 500 g dm^−3^. The dose recommended by manufacturer is 1.0 dm^3^ ha^−1^. It is a selective herbicide with systemic action, which is applied foliarly. It comes in the form of a concentrated suspension for dilution with water.

Sulcotrek 500 SC was produced by Adama Agan Ltd. (Ashdod, Israel), and it was registered in Poland in 2017. It is a selective herbicide with systemic action in the form of a concentrated suspension for dilution with water. It contains two active ingredients: sulcotrione in amount of 173 g dm^−3^ and terbuthylazine in amount of 327 g dm^−3^. The manufacturer recommends using this product at a dose of 2.0 dm^3^ ha^−1^.

### 4.4. Conducting Microbiological Analyses of Soil

Determination of the abundance of organotrophic bacteria, actinobacteria, and fungi was performed in 4 replicates for each soil sample using the serial dilution method according to the procedure described in paper by Kucharski et al. [64] and Borowik et al. [65]. Microorganism cultures were incubated for 10 days in a thermostat at 28 °C. The grown colonies of microorganisms were counted each day using a colony counter, giving their number in cfu kg^−1^ d.m. soil. Based on abundance of above-mentioned microorganisms, colony development (CD) index [66] and ecophysiological diversity (EP) index of microorganisms [67] were calculated.

DNA was isolated from soil without herbicides and with herbicides added at doses 10-fold higher the manufacturer’s recommended, using the Genomic Mini AX Bacteria+ kit (A&A Biotechnology, Gdansk, Poland). Amplification of bacterial 16S rRNA and fungal ITS gene fragment was carried out using universal primers 1055F and 1392R. The procedure for performing DNA isolation and conditions for conducting PCR reaction are described in work by Ferris et al. [68]. Sequencing of genetic material of bacteria was performed based on the V3–V4 hypervariable region of 16S rRNA gene, while fungi were sequenced based on ITS1 region using the Illumina MiSeq v3 instrument (2 × 300 nt) (Illumina, San Diego, CA, USA). Specific primers, 341F and 785R, were used to amplify selected bacterial region, while specific primers, ITS1FI2 and 5.8S, were used to amplify fungi. The obtained sequences of bacteria and fungi were subjected to bioinformatics analysis. Sequencing and bioinformatic analysis was performed by an external company (Genomed S.A., Warsaw, Poland).

### 4.5. Conducting Enzymatic Analyses of Soil

Biochemical analyses of soil were performed in 3 replicates for each combination. Dehydrogenases activity was determined according to Öhlinger procedure [69], catalase—according to Johnson and Temple [70], and alkaline phosphatase, acid phosphatase, *β*-glucosidase, arylsulfatase, and urease according to Alef and Nannipieri [71]. Enzyme activity was determined on a Perkin Elmer Lambda 25 spectrophotometer (Woburn, MA, USA), with exception of catalase, whose activity was determined via titration in the presence of potassium permanganate. A detailed procedure for performing determination of soil enzyme activity is given in work by Baćmaga et al. [72] and Wyszkowska et al. [73].

### 4.6. Conducting Statistical and Bioinformatics Analyses

The results were statistically analyzed at a significance level of *p* = 0.05 using Statistica 13.3 software [74], using multivariate ANOVA tests. Homogeneous groups were developed using Tukey’s post hoc test. Dominant phylum of bacteria and fungi was presented using principal component analysis (PCA). The herbicide impact index (IF) on number of microorganisms and enzyme activity was determined using formula given in the work of Borowik et al. [75]. If IF = 1, it means 100% stimulation of herbicides on analyzed parameter, IF = −1, it means 100% inhibition, IF = 0 means no effect. On basis of plant dry matter yield and the length of aboveground parts, tolerance index (TI) of maize (*Zea mays* L.) was also calculated according to formula presented in the work of Boros et al. [76].

Metagenomic data on bacterial and fungal families were statistically analyzed using the G-test (w/Yates’) and Fisher’s test using STAMP 2.1.3 software [77]. Dominant classes of bacteria and fungi are presented in form of a circular diagram made with Circos 0.68 package [78], and dominant orders and genus of bacteria and fungi are presented in form of a heat map using the RStudio v1.2.5033 software [79], the gplots library version 3.1.3 [80], and the v3.6.2 system [81]. Unique and common genera of bacteria and fungi were also presented in form of a Venn diagram using InteractiVenn software (http://www.interactivenn.net/, accessed on 24 July 2023) [82]. Metagenomic data at all taxonomic levels were presented for OTUs ≥ 1% of sequences obtained.

## 5. Conclusions

Herbicides applied both at the dose recommended by the manufacturer and at a dose 10-fold higher stimulated the proliferation of organotrophic bacteria and the activity of arylsulfatase and acid phosphatase, while causing inhibition of urease activity. They increased the number of bacteria belonging to the phyla, *Actinobacteriota* and *Proteobacteria*, which colonized the soil most numerously. In the case of fungi, there was a decrease in number of the phylum, *Ascomycota*, under the influence of Sulcogan 300 SC, and an increase under the influence of Tezosar 500 SC and Sulcotrek 500 SC. The number of *Mortierellomycota* fungi decreased after the application of Sulcogan 300 SC and Tezosar 500 SC, and increased in soil with Sulcotrek 500 SC. All tested herbicides inhibited the growth of fungi belonging to the phylum, *Basidiomycota*. The maize proved tolerant to the herbicides applied to the soil, as evidenced by an increase in the tolerance index (TI) and the leaf greenness index (SPAD) values of the plants. This research is necessary in the search for better and new solutions in restoring soil homeostasis, and, thorough an understanding of the soil microbiome in the presence of Sulcogan 300 SC, Tezosar 500 SC, and Sulcotrek 500 SC herbicides, it can serve as a suitable tool for the bioaugmnetation of soils contaminated with these preparations. The microorganisms that may possess the ability to inactivate these herbicides are *Lysobacter* and *Sphingobium*, unique in soil with Sulcogan 500 SC, and *Candidatus_Solibacter* and *Haliangium*, univalent in soil with Sulcotrek 500 SC, as well as *Trichaoderma* fungi, present in soil with the tested herbicides.

## Figures and Tables

**Table 4:** Index of influence (IF) of herbicides on soil enzyme activity.

Object	Deh	Cat	Pal	Pac	Glu	Aryl	Ure
SulRD	−0.142 ± 0.012 ^b^	0.003 ± 0.008 ^a^	−0.044 ± 0.059 ^c^	0.078 ± 0.006 ^e^	−0.015 ± 0.011 ^a^	0.379 ± 0.000 ^b^	−0.233 ± 0.016 ^a^
Sul10RD	−0.155 ± 0.010 ^b^	−0.049 ± 0.025 ^b^	−0.220 ± 0.008 ^d^	0.221 ± 0.004 ^bc^	0.011 ± 0.001 ^a^	0.486 ± 0.000 ^a^	−0.340 ± 0.000 ^c^
TezRD	−0.107 ± 0.005 ^ab^	0.001 ± 0.000 ^a^	0.160 ± 0.050 ^b^	0.298 ± 0.008 ^a^	0.004 ± 0.020 ^a^	0.197 ± 0.034 ^c^	−0.339 ± 0.000 ^c^
Tez10RD	−0.081 ± 0.024 ^a^	0.037 ± 0.000 ^a^	0.447 ± 0.046 ^a^	0.205 ± 0.005 ^c^	−0.070 ± 0.001 ^b^	0.151 ± 0.034 ^cd^	−0.362 ± 0.016 ^c^
SucRD	−0.112 ± 0.002 ^ab^	0.026 ± 0.000 ^a^	0.548 ± 0.008 ^a^	0.155 ± 0.012 ^d^	0.019 ± 0.032 ^a^	0.203 ± 0.034 ^c^	−0.278 ± 0.016 ^b^
Suc10RD	−0.079 ± 0.027 ^a^	−0.051 ± 0.016 ^b^	−0.073 ± 0.021 ^c^	0.244 ± 0.005 ^b^	0.009 ± 0.001 ^a^	0.104 ± 0.017 ^d^	−0.510 ± 0.016 ^d^

Sul—Sulcogan 300 SC; Tez—Tezosar 500 SC; Suc—Sulcotrek 500 SC; RD—dose recommended by the manufacturer; 10RD—dose 10-fold higher than recommended by the manufacturer; Deh—dehydrogenases, Cat—catalase, Pal—alkaline phosphatase, Pac—acid phosphatase, Glu—*β*-glucosidase, Aryl—arylsulfatase, Ure—urease; ±—standard deviation. Homogeneous groups denoted by letters (^a–e^) were calculated separately for each enzyme.

## Data Availability

Data are available by contacting the authors.

## Supplementary Materials

The following supporting information can be downloaded at https://www.mdpi.com/article/10.3390/ijms241914469/s1.
