# Supplementary material for: Bacteria, Fungi, and Enzymes in Soil Treated with Sulcotrione and Terbuthylazine
Source: Int J Mol Sci. 2023 Sep 23;24(19):14469. doi: 10.3390/ijms241914469 (PMC10572980; doi:10.3390/ijms241914469)
Supplement: Supplementary file 1 [file ijms-24-14469-s001.zip › ijms-2614132-supplementary.pdf]

International Journal of *Molecular Sciences*

MDPI

Supplementary material

Bacteria, Fungi, and Enzymes in Soil Treated with Sulcotrione and Terbuthylazine

Małgorzata Baćmaga , Jadwiga Wyszkowska*, Jan Kucharski and Agata Borowik

Department of Soil Science and Microbiology, Faculty of Agriculture and Forestry, University of Warmia and Mazury in Olsztyn, 10-719 Olsztyn, Poland; m.bacmaga@uwm.edu.pl (M.B.); agata.borowik@uwm.edu.pl (A.B.); jan.kucharski@uwm.edu.pl (J.K.)

* Correspondence: jadwiga.wyszkowska@uwm.edu.pl

Table S1. Effect of herbicides on abundance of soil microorganisms, cfu 10^n kg^{-1} d.m. soil.

Object	Org × 10^{10}	Act × 10^{10}	Fun × 10^8
C	1.328±0.055^{d}	1.438±0.101^{c}	0.692±0.009^{bc}
SulRD	1.745±0.133^{ab}	1.674±0.038^{b}	0.710±0.004^{b}
Sul10RD	1.427±0.103^{c}	1.476±0.041^{c}	0.571±0.016^{d}
TezRD	1.888±0.163^{a}	1.839±0.111^{ab}	0.868±0.022^{a}
Tez10RD	1.521±0.098^{bc}	1.253±0.065^{d}	0.620±0.020^{cd}
SucRD	1.782±0.055^{ab}	1.244±0.123^{d}	0.752±0.092^{b}
Suc10RD	1.982±0.016^{a}	1.966±0.010^{a}	0.540±0.016^{d}

C—control soil; Sul—Sulcogan 300 SC; Tez—Tezosar 500 SC; Suc—Sulcotrek 500 SC; RD—dose recomended by the manufacturer; 10RD— dose 10-fold higher than recommended by the manufacturer; Org—organotrophic bacteria; Act—actinobacteria; Fun—fungi; ±—standard deviation. Homogeneous groups denoted by letters ($^{a-d}$) were calculated separately for each group of microorganisms.

Table S2. Effect of herbicides on soil enzyme activity, kg^{-1} d. m. soil h^{-1}.

Object	Deh	Cat	Pal	Pac	Glu	Aryl	Ure
	μmol TFF	mol O_2	mmol PNP				mmol N-NH_4
C	19.918±1.376^{a}	0.357±0.004^{a}	0.335±0.016^{c}	2.246±0.079^{e}	0.248±0.008^{a}	0.165±0.014^{c}	0.859±0.017^{a}
SulRD	17.097±0.300^{b}	0.358±0.004^{a}	0.320±0.024^{c}	2.421±0.015^{e}	0.245±0.003^{a}	0.227±0.002^{a}	0.659±0.017^{b}
Sul10RD	16.827±0.237^{b}	0.340±0.011^{b}	0.261±0.003^{d}	2.743±0.010^{b}	0.251±0.003^{a}	0.245±0.003^{a}	0.567±0.003^{c}
TezRD	17.779±0.119^{b}	0.358±0.001^{a}	0.389±0.021^{b}	2.915±0.022^{a}	0.249±0.006^{a}	0.197±0.007^{b}	0.567±0.003^{c}
Tez10RD	18.296±0.590^{ab}	0.370±0.001^{a}	0.485±0.019^{a}	2.706±0.013^{b}	0.231±0.002^{b}	0.190±0.007^{b}	0.548±0.017^{c}
SucRD	17.691±0.060^{b}	0.367±0.001^{a}	0.519±0.003^{a}	2.595±0.032^{c}	0.253±0.010^{a}	0.198±0.007^{b}	0.620±0.017^{b}
Suc10RD	18.353±0.658^{ab}	0.339±0.007^{b}	0.311±0.009^{c}	2.794±0.013^{b}	0.250±0.003^{a}	0.182±0.003^{bc}	0.421±0.017^{d}

C—control soil; Sul—Sulcogan 300 SC; Tez—Tezosar 500 SC; Suc—Sulcotrek 500 SC; RD—dose recomended by the manufacturer; 10RD— dose 10-fold higher than recommended by the manufacturer; Deh—dehydrogenases, Cat—catalase, Pal—alkaline phosphatase, Pac—acid phosphatase, Glu—*β*-glucosidase, Aryl—arylsulfatase, Ure—urease; ±—standard deviation. Homogeneous groups denoted by letters ($^{a-e}$) were calculated separately for each enzyme.

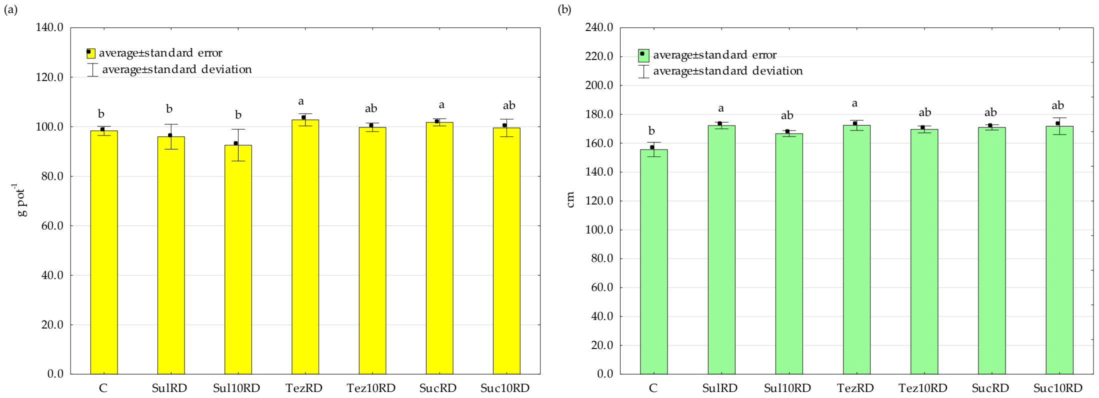

Figure S1. Effect of herbicides on (a) dry matter yield, and (b) length of aboveground parts of *Zea mays* L. C—control soil; Sul—Sulcogan 300 SC; Tez—Tezosar 500 SC; Suc—Sulcotrek 500 SC; RD—dose recomended by the manufacturer; 10RD— dose 10-fold higher than recommended by the manufacturer. Homogeneous groups denoted with letters ($^{a-b}$).
